# Host specificity in a diverse Neotropical tick community: an assessment using quantitative network analysis and host phylogeny

**DOI:** 10.1186/s13071-016-1655-6

**Published:** 2016-06-29

**Authors:** Helen J. Esser, Edward Allen Herre, Nico Blüthgen, Jose R. Loaiza, Sergio E. Bermúdez, Patrick A. Jansen

**Affiliations:** Smithsonian Tropical Research Institute, Balboa, Ancon, Panama; Department of Environmental Sciences, Wageningen University, Wageningen, The Netherlands; Department of Biology, Technische Universität Darmstadt, Darmstadt, Germany; Centro de Biodiversidad y Descubrimiento de Drogas, Instituto de Investigaciones Científicas y Servicios de Alta Tecnología, Clayton, Panamá, República de Panamá; Departamento de Investigación en Entomología Médica, Instituto Conmemorativo Gorgas de Estudios de la Salud, Panamá, República de Panamá

**Keywords:** Host-parasite associations, Host relationships, Vector, Interaction network, Mean pairwise phylogenetic distance, Ixodidae, Argasidae, Panama

## Abstract

**Background:**

Host specificity is a fundamental determinant of tick population and pathogen transmission dynamics, and therefore has important implications for human health. Tick host specificity is expected to be particularly high in the tropics, where communities of ticks, hosts and pathogens are most diverse. Yet the degree to which tropical tick species are host-specific remains poorly understood. Combining new field data with published records, we assessed the specificity of tick-host associations in Panama, a diverse Neotropical region.

**Methods:**

The resulting dataset includes 5,298 adult ticks belonging to 41 species of eight genera that were directly collected from 68 vertebrate host species of 17 orders. We considered three important aspects of tick host specificity: (i) the relative ecological importance of each host species (structural specificity); (ii) relatedness among host species (phylogenetic specificity); and (iii) spatial scale-dependence of tick-host relationships (geographical specificity). Applying quantitative network analyses and phylogenetic tools with null model comparisons, we assessed the structural and phylogenetic specificity across three spatial scales, ranging from central Panama to countrywide. Further, we tested whether species-rich tick genera parasitized a wider variety of hosts than species-poor genera, as expected when ticks specialize on different host species.

**Results:**

Most tick species showed high structural and/or phylogenetic specificity in the adult stage. However, after correcting for sampling effort, we found little support for geographical specificity. Across the three scales, adult ticks tended to be specific to a limited number of host species that were phylogenetically closely related. These host species in turn, were parasitized by tick species from distinct genera, suggesting switching among distantly related hosts is common at evolutionary timescales. Further, there was a strong positive relationship between the taxonomic richness of the tick genera and that of their hosts, consistent with distinct tick species being relatively specific to different host species.

**Conclusions:**

Our results indicate that in the adult stage, most ticks in the diverse Neotropical community studied are host specialists. This contrasts with earlier assessments, but agrees with findings from other host-parasite systems. High host specificity in adult ticks implies high susceptibility to local tick-host co-extirpation, limited ability to colonize new habitats and limited potential for interspecific pathogen transmission.

**Electronic supplementary material:**

The online version of this article (doi:10.1186/s13071-016-1655-6) contains supplementary material, which is available to authorized users.

## Background

Host specificity is a fundamental life history trait of parasites that is likely to play a major role in generating and maintaining parasite biodiversity [1, 2]. The degree to which parasites are host-specific is a key determinant of their ability to colonize new host species [3], their geographical range size and local abundance [4, 5], the probability of parasite-host coextinction [6, 7] and the potential routes by which pathogens can be transmitted across vertebrate host taxa, including humans [8]. Hence, quantifying host specificity will help elucidate the ecological and co-evolutionary relationships between parasite and host species that are relevant for human and veterinary medicine, as well as for biodiversity conservation [9, 10].

A group of organisms in which the question of host specificity is particularly important, are ticks (Acari: Ixodida). Ticks are obligatory hematophagous ectoparasites that feed on every class of terrestrial vertebrates throughout the world [11]. They are major vectors of diseases to both humans and livestock, imposing a significant burden on public health and livestock producers [12]. Ticks are especially abundant in the tropical regions, both in species and in numbers [13]. The tropics are also hotspots for vertebrate diversity [14] and hence are rich in potential host species for ticks. Resource specialization has been suggested as an important factor driving the remarkable species richness in these systems [15–17]. Indeed, empirical studies of other host-parasite systems have shown that parasites tend to be more specific in richer host faunas [18, 19]. Further, several features of ticks are predicted to limit their host ranges and select for host specificity (see [1, 8, 20] for a review) and host specificity is therefore expected to be high for tropical tick species.

Relatively few empirical studies have tested this hypothesis, none of which found conclusive evidence that high host specificity in tropical tick species is common. Cumming [21] analyzed a large dataset on African tick-host associations (Ixodidae and Argasidae) and concluded that these ticks showed a continuum in their degree of host specificity, ranging from specialists at the host species-, family-, or order-level to broad host generalists of a wide variety of vertebrate orders. A more recent study on ixodid ticks of mammals in South Africa found, depending on the specificity index used, that ticks showed either very low or a wide diversity of specificity in all life stages [22]. Using the same index, Nava & Guglielmone [23] performed a meta-analysis on Neotropical ixodid ticks and argued that while some tick species are specific at the host genus- or family-level, strict host specificity is uncommon. These previous studies, however, did not correct for host availability or for the likelihood of observing the recorded tick host-use patterns. After accounting for these biases, Wells et al. [24] found little evidence for host specificity in ixodid ticks of small mammals in Borneo. But because host associations of adults, nymphs and larvae were not analyzed independently, stage-specific host specificity could have been missed in that study. Indeed, Espinaze et al. [22] and Nava & Guglielmone [23] found that host specificity differed among life stages, with immature ticks typically being more generalist than their adult conspecifics. Hence, the degree to which different life stages of tropical ticks are host-specific remains poorly understood and further studies are warranted.

The complexity of the tick-host interface requires consideration of at least three different aspects when measuring host specificity. First, structural differences in the distribution of tick populations across vertebrate hosts reflect the relative ecological importance of each exploited host species [25]. For example, two tick species that exploit the same number of host species may differ greatly in the extent to which they use each of these hosts [8, 23]. Secondly, phylogenetic relatedness among host species is another important determinant of evolutionary specialization that is not always considered [26]. Preferred, more frequently parasitized host species may be more closely related to one another than sporadically parasitized host species [25]. Finally, specificity can also be measured as the consistency in host-use across a changing host landscape [25]. A growing number of studies suggest that host specificity in ticks may be spatially scale-dependent; with ticks tending to be host specialists at local scales and host generalists at larger geographical scales (reviewed by [8]). These different aspects of host specificity are known as *structural specificity*, *phylogenetic specificity* and *geographical specificity*, respectively [25], and they may vary markedly among the different life stages of a tick species [13, 23]. To our knowledge, no study has so far considered all these aspects of specificity in tropical tick-host communities.

Here, we investigate the degree to which adult ticks are host-specific in Panama, a diverse Neotropical region supporting over 40 species of ticks. Focusing on adult ticks, we assessed (i) the structural specificity of ticks at both the species- and community-level using quantitative network analyses that control for host availability; (ii) the phylogenetic specificity of ticks by estimating the standardized effect size of the mean pairwise phylogenetic distance of exploited host species; and (iii) the geographical specificity by comparing structural and phylogenetic specificity across three nested spatial scales that ranged from local (central Panama) to countrywide. We applied rarefaction to account for variation in the number of potential host species across the three spatial scales, and used null model comparisons to evaluate the likelihood of observing the recorded tick-host associations. We also tested whether species-rich tick genera parasitized a wider variety of hosts than species-poor genera, as would be expected if tick species have specialized on different host taxa. Lastly, we discuss the associations between ticks and domestic animals as these potentially include new relationships formed over relatively short evolutionary time periods.

## Methods

### Study area

Data were collected throughout Panama, part of the world’s second largest ‘megadiversity hotspot’ for endemic vertebrates [14]. Over forty species of ticks have been reported from Panama, divided over eight genera and two families [27–30]. Panama also has a wide variety of environmental conditions and habitats, ranging from mangrove swamps to tropical forests and from savannahs to the páramo. Elevation ranges from *c*.0–3,500 m. Panama has a tropical moist weather pattern with an average diurnal temperature of 27 °C. Average temperature and humidity are high throughout most of the country, but considerably milder at elevations > 600 m. Rainfall varies both regionally (*c*. 1,750–4,000 mm) and temporally, with a pronounced dry season in the lowlands from January to April [31].

### Data collection

We collected data on host feeding relationships of ticks (Ixodidae and Argasidae) from January 2009 until May 2014. Sampled hosts included wild animals, either live-captured or found as road kills, as well as humans and domestic animals from different environments throughout Panama. We searched the entire body of hosts but only ticks found firmly attached were considered in further analyses. Ticks were preserved in 95 % ethanol and later identified using the taxonomic keys provided by Fairchild et al. [27] and Onofrio et al. [32]. We used the taxonomic criteria of Nava et al. [29] for the *Amblyomma cajennense* species complex, which is represented by *A. mixtum* in Panama. Additional data on ticks and their vertebrate hosts were obtained from published regional monographs (see Additional file 1: Table S1).

Most tick species of the family Ixodidae are characterized by a three-host life-cycle, in which the larvae, nymphs and adults feed from different host individuals that may belong to distinct species [13]. Hence, pooling data on host associations of different tick life stages could confound potential patterns of stage-specific host specificity and such data should therefore be analyzed separately. Unfortunately, the larvae and nymphs of the three-host ticks in Panama (35 out of 37 species of Ixodidae) are notoriously difficult to identify, making earlier records unreliable. Moreover, the immature life stages of several tick species in our dataset remain undescribed [13]. We therefore limited our study to adult ticks and included species of both Ixodidae and Argasidae; the species of the latter family are also generally characterized by possessing multi-host life-cycles [33].

**Fig. 1:**
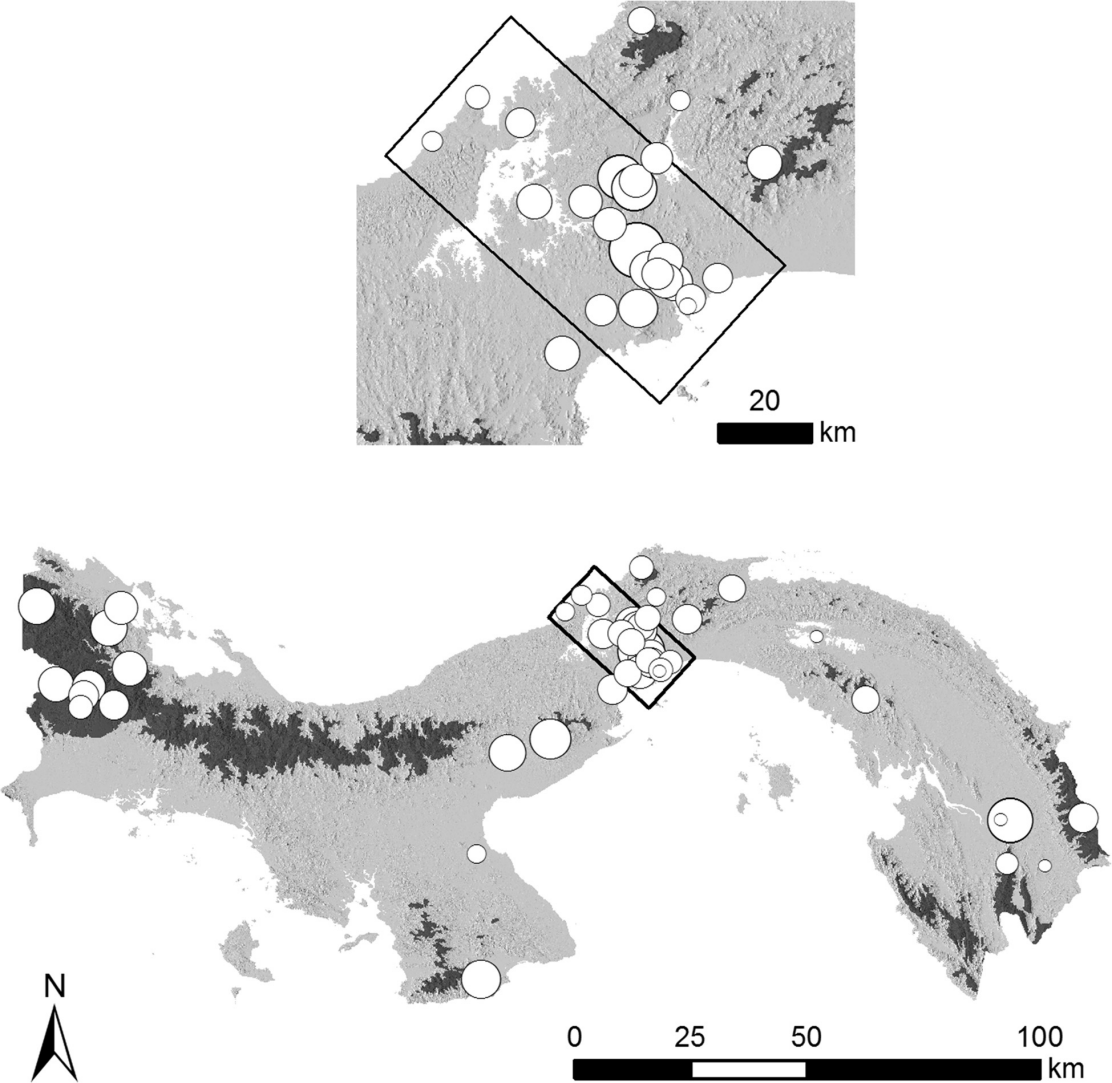
Map of Panama showing the sampling locations across the three spatial scales: large (entire country), intermediate (light grey areas), and small (black box inset). These sampling locations show the minimum coverage as the description of many collection localities retrieved from literature did not allow for a specific placement on the map

The overall dataset included adult tick-host associations from a wide variety of habitats and altitudes collected in over 54 locations throughout the country (Fig. 1). The true coverage is much larger but the description of many collection localities retrieved from the literature did not allow for a specific allocation on the map, even though they could be used for the analysis of geographical specificity (see below). We followed the consensus list of valid tick names as compiled by Guglielmone et al. [34], which recognizes three genera of Argasidae (i.e. *Antricola*, *Argas* and *Ornithodoros*) and five genera of Ixodidae (i.e. *Amblyomma*, *Dermacentor*, *Haemaphysalis*, *Ixodes* and *Rhipicephalus*) for Panama.

### Structural specificity

Indices of host specificity that consider both the number of host species and the relative frequency with which they are exploited, such as those based on the widely used Shannon index in ecology, are excellent for measuring structural host specificity [25]. Here, we used two such metrics: *H*_2_^'^ and *d*_*i*_^'^ [35]. These metrics were developed for the analysis of bipartite networks, a standardized framework for the quantification of ecological specialization [36, 37]. Bipartite networks represent associations (links) between species (nodes) of two trophic levels and are either based on weighted (quantitative) or unweighted (binary) links. The two metrics used here are based on weighted links, i.e. they were calculated using the relative frequencies with which tick-host associations occur (see Additional file 2: Table S1 for formulas). By accounting for variation in the “strength” of the interactions, they provide an ecologically more meaningful measure of host specificity than do metrics based on unweighted links, i.e. presence/absence data [35, 38]. Both indices were calculated using the network-level [39] and species-level analyses [40] tools in the R package ‘bipartite’ [41].

The *H*_2_^'^ index, the standardized two-dimensional Shannon entropy, is a measure of structural specificity of the entire network, henceforward community-level [35]. Values range from 0 for the most generalist community to 1 for the most specialist community. The index increases with deviations of the network’s observed frequency distribution of species interactions from their expected probability distribution. This null distribution of interactions reflects a situation where all species interact with their partners in proportion to their observed frequency totals [35].

The *d*_*i*_^'^ index, the standardized Kullback-Leibler distance, is a measure of structural specificity for each individual node, henceforward species-level [35]. Like *H*_2_^'^, values range from 0 for the most generalist to 1 for the most specialist species. For species *i*, the value of *d*_*i*_^'^ increases with deviations of the observed frequency distributions from a null distribution that assumes that the interactions with species *i* are proportional to overall partner availability. Thus, *d*_*i*_^'^ increases with reciprocal specificity between two partners and hence reflects the “exclusiveness” of species interactions [35].

These two indices take into account what many other host specificity indices do not: resource availability. If not accounted for, estimates of host specificity of ticks that occur in only a few samples will be biased, with rare species being systematically classified as more specific [42, 43]. The *H*_2_^'^ and *d*_*i*_^'^ indices do not suffer from this classical artefact since the use of rare resources (i.e. host species) is not given the same weight as the use of common ones. Thus, these indices are able to discriminate species with strong host preferences from those using available host species simply in proportion to their occurrence in the environment.

However, Dormann et al*.* ([39] and references therein) showed that most metrics, including those based on weighted links, are affected by network dimensions (i.e. number of species) and sampling intensity (i.e. total observation records per species). Observed metric estimates should therefore be evaluated against expectations based on null models that control for these network properties [36, 39]. Here, we used two such null models, each with 1,000 replicates, to test whether the observed estimates deviated significantly from what would be expected by chance.

Null model I was based on an algorithm developed by Patefield [44], which randomly redistributes the interactions across all species in the matrix while maintaining column and row totals identical to those of the observed matrix. This algorithm is analogous to most re-sampling-based contingency table tests such as *χ*^2^ or Fisher’s exact test [39] and is implemented in the R package ‘*bipartite*’ as function ‘*r2dtable*’ [41]. By constraining the marginal sums, this null model corrects for uneven numbers of species observation records [36].

Null model II was based on an algorithm developed by Vázquez et al. [45], which redistributes the interactions only across those species that were actually observed to interact, thereby maintaining connectance. This algorithm is implemented in the R package ‘*bipartite*’ as ‘*vaznull*’ [41]. By constraining the realized links of the original network, it takes into account that unrealized connections between certain tick and host species may in fact represent life-history restrictions, i.e. ‘forbidden links’. These forbidden links may arise from a lack of host availability, such as non-overlap of tick and host habitat in space or time, but may also result from host avoidance. Hence, null model II can be regarded as very constrained in comparison with null model I.

### Phylogenetic specificity

Closely related species tend to share similar biological, behavioral and physiological traits [26]. Hence, the more phylogenetically related a given set of host species, the more likely they should be to share the same parasite species. In comparative analysis, this is similar to the problem of non-independence of species [46]. We used a widely employed method to assess relatedness among host species in each tick species’ diet: the mean phylogenetic distance (MPD) between each pair of parasitized host species [47, 48]. We used a taxonomic classification with 19 hierarchical levels above species (see Additional file 3: Figure S1). Branch lengths were set to unity and we weighted the MPD by the number of tick-host associations. This method is fairly independent from species richness and therefore from sampling effort [26, 48].

However, the extent to which parasitized host species represent a non-random selection from the total host community cannot generally be assessed using raw MPD values [48]. We therefore calculated standardized effect sizes of the MPD values (SES_MPD_) to evaluate whether observed host relationships deviated from what would be expected based on the relatedness of the available host species. The SES_MPD_ is basically a *Z*-score, which describes the difference between the observed MPD and the MPD expected under a null model, divided by the standard deviation of the MPD in the null data. This approach is equivalent to -1 times the Net Relatedness or Nearest Relative Index (NRI) that is widely used in community ecology and has a similar interpretation [26, 49]. The null model that we used here randomizes the names of the host species on the terminal branches of the phylogeny, so that the distribution of the branches remains intact. This null model is implemented in the R package ‘*picante*’ as “taxa.labels” [49]. Positive SES values indicate greater phylogenetic distance among parasitized host species than expected by chance, whereas negative SES values indicate small phylogenetic distances, i.e. high phylogenetic specificity.

### Geographical specificity

To assess structural and phylogenetic host specificity in geographical space, we subsetted the total dataset twice, yielding separate datasets on tick-host associations for three scales: (i) the entire country of Panama (*c*.74,340 km^2^), including a wide variety of natural and anthropogenic habitats ranging from lowlands to highlands up to 3,000 m; (ii) the lowlands of Panama (*c*.59,710 km^2^), including a variety of natural and anthropogenic habitats up to 600 m; and (iii) central Panama (*c*.2,178 km^2^), including an area of 20 km on either side of the Panama Canal, most of which lies below 300 m with a uniform temperature and humidity [50]. Henceforward, these three spatial scales will be referred to as “large”, “intermediate” and “small”, respectively (see Additional file 1: Table S1 for more details).

While we used null models to compare the patterns within the species data matrix, we need to consider for our comparison across the three spatial scales that the local dataset is nested in the regional one, and the regional is nested in the nation-wide data. Hence, our tick-host community matrices are additive, so that resource potential increases with scale. If not corrected for this sampling bias, a decline in host specificity with increasing spatial scale (*sensu* McCoy et al. [8]) may simply arise due to a larger number of available host species [42]. For a meaningful comparison of structural and phylogenetic specificity across the three scales, we therefore rarefied the largest two matrices so that their total number of interactions was identical to the smallest matrix. Using the ‘*sample*’ command in R with 1,000 randomizations, we resampled the entries of the matrix with a probability for sampling each link given by the proportion of its link strength (see Additional file 2: Table S1). All analyses were carried out with the R statistical software, version 3.2.4 [51].

### Richness relationships

If tick species specialize on different host taxa, then more species-rich tick genera should parasitize a wider variety of hosts than species-poor genera. However, the observed number of host species is likely to be an underestimate since species richness is strongly affected by sampling effort. We corrected for biases arising from the undersampling of rare host species by computing the Chao1 index, an abundance-based estimator for asymptotic species richness [52], using EstimateS version 9.1.0 [53]. We used Spearman’s rho (ρ) to test the prediction that a positive relationship exists between generic tick species richness and generic Chao1 estimates of total host species richness.

Because *d*_*i*_^'^ and MPD are more sophisticated measures of host specificity than the basic number of host species, we also tested for the relationship between these two indices and generic tick species richness. If most tick species show high structural specificity towards different species of hosts, then generic *d*_*i*_^'^ estimates should be higher for species-poor tick genera than for species-rich tick genera. Hence, we expected *d*_*i*_^'^ to decline with generic tick species richness. In contrast, if most tick species show high phylogenetic specificity towards different species of hosts, then MPD estimates should be lower for species-poor tick genera than for species-rich tick genera. Hence, we expected MPD estimates to increase with generic tick species richness.

## Results

### Structural specificity

**Fig. 2:**
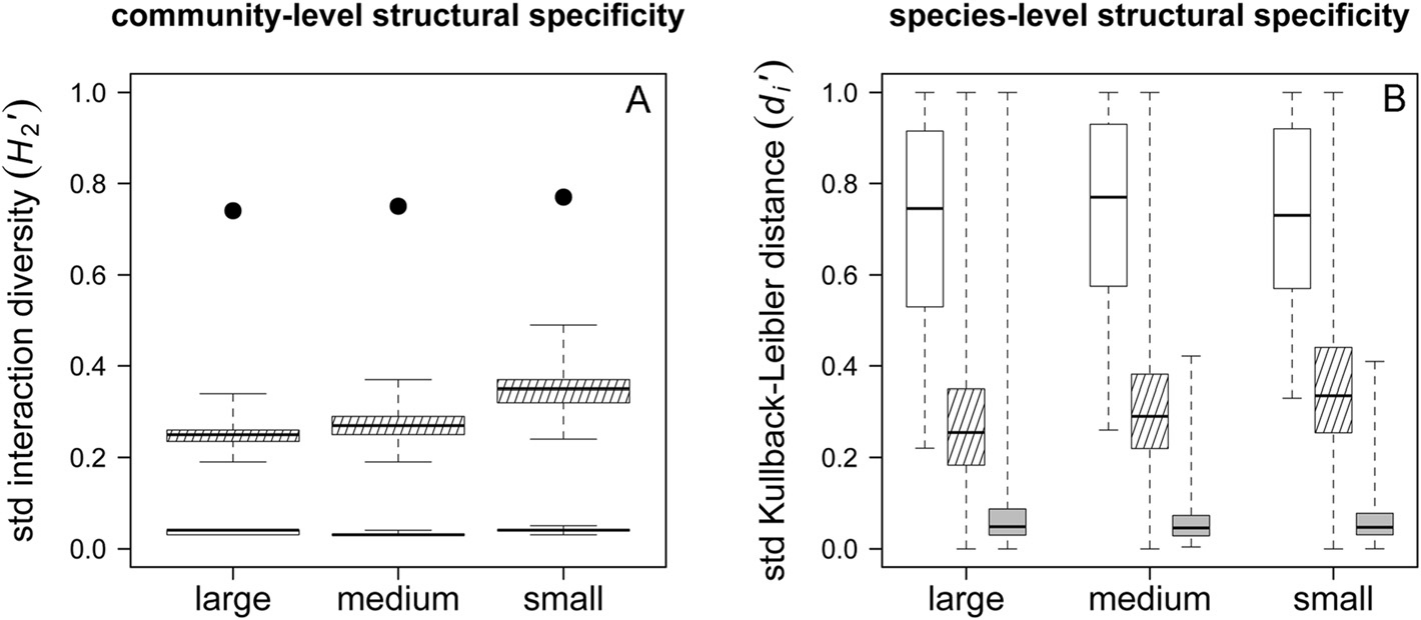
Observed *vs* null model estimates of structural host specificity. Observed estimates for **a** community-level specialization *H*_2_^'^ (*black dots*) and **b** species-level specialization *d*_*i*_^'^ (*white box plots*) are much larger than estimates predicted by null model I (*grey box plots*) and null model II (*dashed box plots*) for each spatial scale (large, intermediate, small). Plot whiskers extend from minimum to maximum estimates

Structural specificity of the entire network was high for each spatial scale (large: *H*_2_^'^ = 0.74; intermediate: *H*_2_^'^ = 0.75; small: *H*_2_^'^ = 0.77). Significance was assessed by determining the proportion of randomized estimates (*n* = 1,000) that was equal to or greater than the observed *H*_2_^'^ estimate. For each spatial scale, the observed *H*_2_^'^ estimate was significantly larger than predicted by each of the two null models (*P* = 0), indicating high structural specificity of tick-host communities (Fig. 2a).

Structural specificity values at the species-level (*d*_*i*_^'^) ranged from 0.22 to 1.00 (median 0.76) at the large scale, from 0.26 to 1.00 (median 0.77) at the intermediate scale, and from 0.33 to 1.00 (median 0.73) at the small scale (Fig. 2b). Significance was assessed for each tick species by determining the proportion of randomized estimates (*n* = 1,000) that was equal to or greater than the observed *d*_*i*_^'^ estimate. With a single exception, all observed *d*_*i*_^'^ estimates were significantly higher than predicted by null model I for each spatial scale (Table 1). Compared to the more constrained null model II however, observed *d*_*i*_^'^ estimates were significantly higher for 30 out of 41 tick species at the large scale, 21 out of 28 tick species at the intermediate scale, and 15 out of 25 tick species at the small scale.

**Table 1:** Observed values for structural (*d*_*i*_^'^) and phylogenetic (SES_MPD_) specificity at the species-level. Values are shown for each spatial scale. Significance as assessed by each null model (NM I, II, III) is given as ***P < 0.001; **P < 0.01; *P < 0.05, or ns (not significant)

	Large					Intermediate					Small				
	*d*_*i*_^'^	NM I^a^	NM II^b^	SES_MP_	NMIII^c^	*d*_*i*_^'^	NM I^a^	NM II^b^	SES_MPD_	NMIII^c^	*d*_*i*_^'^	NM I^a^	NM II^b^	SES_MPD_	NMIII^c^
*Amblyomma* spp.															
*A. auricularium*	0.88	***	***	-2.02	*	0.89	***	***	-1.91	*	0.97	***	**	-1.83	ns
*A. calcaratum*	0.45	***	ns	-2.32	*	0.41	***	ns	-2.00	*	0.66	***	ns	-2.02	*
*A. coelebs*	0.42	***	ns	-2.63	**	0.41	***	ns	0	***	0.49	***	ns	0	***
*A. dissimile*	0.95	***	**	-2.40	**	0.94	***	***	-2.40	**	0.94	***	***	-2.59	**
*A. geayi*	0.87	***	***	-2.60	***	0.87	***	***	-2.58	***	0.85	***	***	-2.48	***
*A. longirostre*	1.00	***	*	0	***	1.00	***	*	0	***	1.00	***	*	0	***
*A. mixtum*	0.40	***	ns	-2.67	**	0.42	***	ns	-2.48	*	0.40	***	ns	-2.75	**
*A. naponense*	0.61	***	*	-0.72	ns	0.61	***	*	-0.64	ns	0.57	***	ns	-0.72	ns
*A. nodosum*	0.92	***	***	-2.58	**	0.92	***	***	-2.48	**	0.87	***	*	0	***
*A. oblongoguttatum*	0.22	***	ns	-3.24	**	0.26	***	ns	-2.89	**	0.33	***	ns	-2.59	**
*A. ovale*	0.58	***	***	-2.04	*	0.65	***	***	-2.08	**	0.67	***	**	-1.84	*
*A. pacae*	0.82	***	*	-2.36	*	1.00	***	*	0	***	1.00	***	*	0	***
*A. parvum*	0.37	***	ns	-1.58	ns	0.36	***	ns	-1.49	ns	0.37	***	ns	-0.35	ns
*A. pecarium*	0.76	***	**	0	***	0.76	***	*	0	***	0.81	***	*	0	***
*A. pictum*	0.54	*	ns	0	***	–	–	–	–	–	–	–	–	–	–
*A. sabanerae*	0.94	***	**	-2.65	**	0.94	***	**	-2.64	**	0.93	***	**	-2.68	**
*A. tapirellum*	0.62	***	***	-2.21	*	0.67	***	***	-2.18	**	0.62	***	*	-1.40	ns
*A. varium*	0.64	***	*	-2.46	*	0.61	***	*	-2.45	**	0.56	***	ns	-2.34	*
*Antricola *spp.															
*A. mexicanus*	1.00	***	*	0	***	1.00	***	*	0	***	–	–	–	–	–
*Argas* spp.															
*A. persicus*	0.99	***	**	0	***	1.00	***	*	0	***	–	–	–	–	–
*Dermacentor* spp.															
*D. imitans*	0.47	***	ns	-0.73	ns	–	–	–	–	–	–	–	–	–	–
*D. latus*	0.47	***	ns	-1.17	ns	–	–	–	–	–	–	–	–	–	–
*D. nitens*	0.73	***	**	-1.69	*	0.58	***	ns	-1.61	*	0.60	***	ns	-1.71	*
*D. panamensis*	0.91	***	*	0	***	–	–	–	–	–	–	–	–	–	–
*Haemaphysalis* spp.															
*H. juxtakochi*	0.57	***	*	-1.97	*	0.57	***	*	-2.08	**	0.59	***	ns	-2.12	*
*H. leporispalustris*	0.98	***	**	0	***	1.00	***	*	0	***	–	–	–	–	–
*Ixodes* spp.															
*I. affinis*	0.79	***	*	-1.90	*	0.78	***	*	-1.32	ns	0.80	***	*	-1.12	ns
*I. auritulus*	1.00	***	*	-1.39	ns	–	–	–	–	–	–	–	–	–	–
*I. bequearti*	1.00	***	**		***	–	–	–	–	–	–	–	–	–	–
*I. boliviensis*	0.46	***	*	-1.52	ns	–	–	–	–	–	–	–	–	–	–
*I. lasallei*	0.83	***	*	-2.36	**	–	–	–	–	–	–	–	–	–	–
*I. luciae*	0.94	***	**	-3.04	***	0.89	***	*	-2.80	***	0.96	***	*	-2.84	***
*I. pomerantzi*	0.58	***	ns	0	***	–	–	–	–	–	–	–	–	–	–
*I. rubidus*	0.83	***	*	-3.74	***	–	–	–	–	–	–	–	–	–	–
*I. tapirus*	0.22	ns	ns	0	***	–	–	–	–	–	–	–	–	–	–
*I. tiptoni*	1.00	***	*	0	***	–	–	–	–	–	–	–	–	–	–
*I. venezuelensis*	1.00	***	*	-2.42	**	–	–	–	–	–	–	–	–	–	–
*Ornithodoros* spp.															
*O. puertoricensis*	0.70	***	ns	0.65	ns	0.79	***	*	0.69	ns	0.92	***	*	0.65	ns
*O. rudis*	0.52	***	ns	0	***	0.52	***	ns	0	***	0.51	***	ns	0	***
*Rhipicephalus* spp.															
*R. microplus*	0.79	***	**	-2.69	**	0.84	***	***	-2.60	**	0.85	***	***	-2.69	**
*R. sanguineus*	0.65	***	***	-1.05	ns	0.72	***	***	-0.78	ns	0.73	***	**	-0.45	ns

^a^NM I, null model I, Patefield algorithm, significance for *d*_*i*_^'^

^b^NM II, null model II, Vaznull algorithm, significance for *d*_*i*_^'^

^c^NM III, randomization of taxa labels, significance for SES_MP_

*Abbreviation*: *ns* not significant

While comparisons with null model I provide an upper bound estimate of the number of specialist tick species, comparisons with null model II provide a lower bound estimate. This is because null model I assumes that all host species in the dataset are available to each tick species, whereas null model II assumes that any unrealized connection between a tick and host species represents a forbidden link. Since some forbidden links may actually reflect host avoidance rather than a lack of host availability, part of the tick species that appear to be host generalists under null model II are in fact host specialists that do not discriminate among the, sometimes quite limited, number of host species they do parasitize. This may be true for several tick species that were almost exclusively collected from a single host species (e.g. *Amblyomma coelebs* and *Dermacentor latus* on Baird’s tapir, *Amblyomma naponense* and *Dermacentor imitans* on collared peccary, *Dermacentor nitens* on horse), or that were abundant on only a small number of host species (e.g. *Amblyomma calcaratum* on anteaters, *Amblyomma varium* on sloths). Overall, these results suggest high structural specificity at the host species-, family-, or order-level during the adult life stage of most tick species in Panama.

### Phylogenetic specificity

**Fig. 3:**
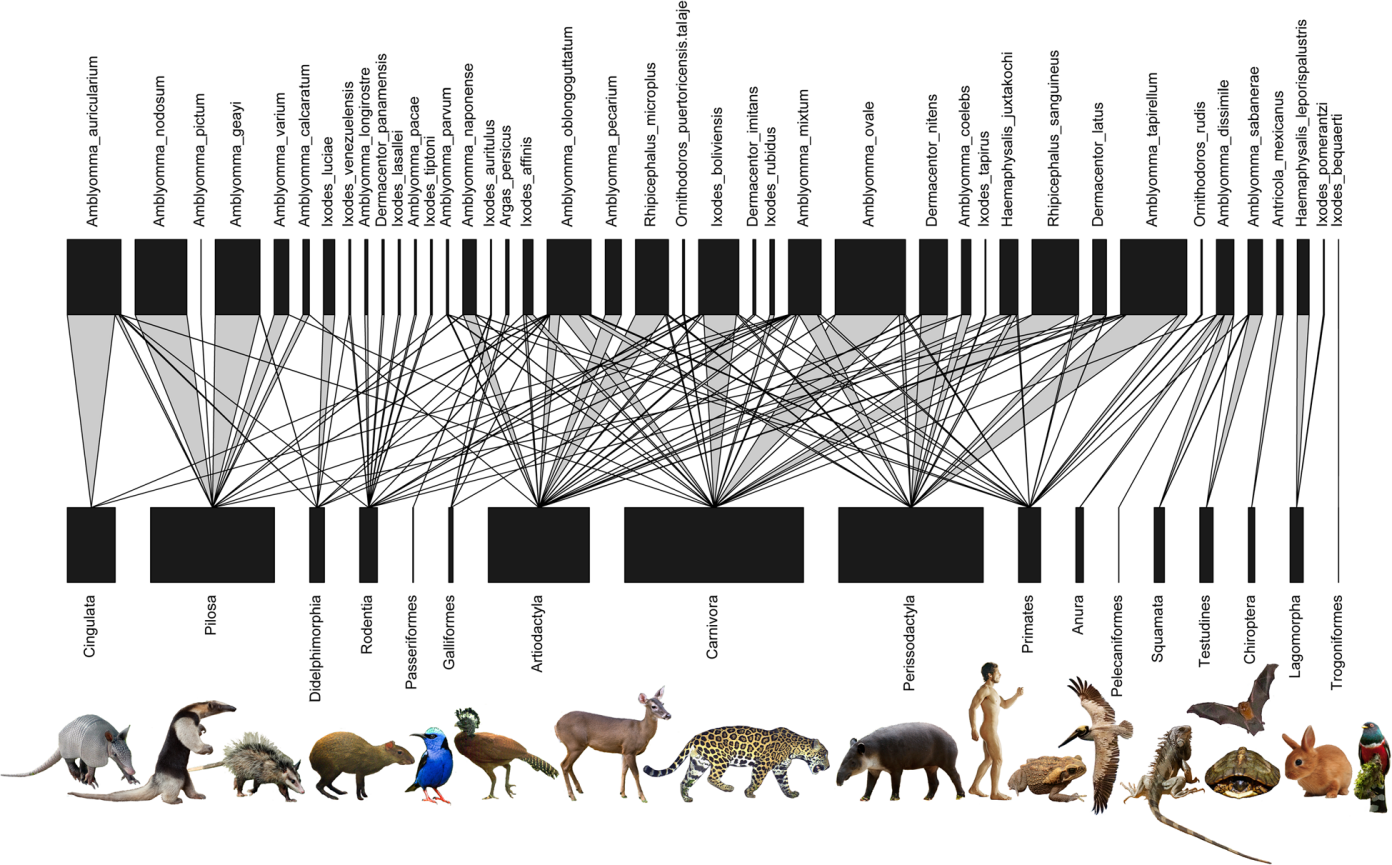
Quantitative tick-host interaction network. Relationships between the tick species of Panama and their vertebrate hosts as visualized by a bipartite network. Host species are pooled to the taxonomic level of vertebrate order for clarity. Nodes (*black*) represent species and links (*grey*) correspond to species interactions. Variation in interaction frequencies are reflected by the width of the links. The network is arranged such that it shows minimal crossings of interactions, which allows for easier interpretation

For 29 out of 41 tick species, over 90 % of the collection records came from a single vertebrate order, and 12 tick species were each associated with a single vertebrate species. This suggests that many tick species in Panama are associated with phylogenetically closely related host species during the adult life stage. Indeed, the SES_MPD_ estimates showed that at the large scale, 33 out of 41 species were phylogenetically more host-specific than expected by chance. At the intermediate scale this was true for 23 out of 28 tick species, and at the small scale, 18 out of 25 species of ticks showed significant phylogenetic specificity (Table 1). Phylogenetic specificity was found at the level of host species (e.g. *Haemaphysalis leporispalustris* on forest rabbit), host family (e.g. *Amblyomma nodosum* on anteaters), and host order (e.g. *Ixodes rubidus* on carnivores), although some tick species parasitized several host orders (e.g. *Amblyomma dissimile* on amphibians and reptiles, *Haemaphysalis juxtakochi* on odd- and even-toed ungulates). Interestingly, while most ticks tended to feed from phylogenetically closely-related host species, these hosts themselves were parasitized by tick species from distinct genera (Fig. 3).

### Geographical specificity

Overall, we did not find strong evidence for scaling of host specificity with geographical space. While structural specificity at the community-level (*H*_2_^'^) declined marginally with increasing scale, it remained high for each spatial scale and these values were not affected by rarefaction. Similarly, structural specificity at the species-level (*d*_*i*_^'^) was high for each spatial scale, with negligible effects of rarefaction and no clear trend across the three spatial scales. Four tick species that showed structural specificity at larger scales did not do so at smaller spatial scales when compared to null model II values. One tick species (*Ornithodoros puertoricensis*) showed the opposite trend (Table 1). As a result, the proportion of structural specialists was slightly lower at the smallest scale. Finally, no major changes were observed for phylogenetic specificity across the three spatial scales. With only three exceptions, tick species whose MPD values were significant at larger scales, were also significant at smaller spatial scales when recorded. The proportion of phylogenetic specialists was slightly lower at the smallest scale.

### Richness relationships

There was a strong, positive correlation between the number of species within each tick genus and the estimated total number of host species (Chao1) parasitized by that genus (Spearman’s *ρ* = 0.93, *P* = 0.001). Likewise, we found a significant positive correlation between generic MPD (phylogenetic specificity at the tick genus-level) and generic tick species richness (Spearman’s *ρ* = 0.83, *P* = 0.011). There was a significant negative correlation between generic *d*_*i*_^'^ (structural specificity at the tick genus-level) and generic tick species richness (Spearman’s *ρ* = 0.95, *P* < 0.0001). These results suggest that different tick species within the same genus are specific to different host species, both structurally and phylogenetically.

### Relationships with domestic animals

**Fig. 4:**
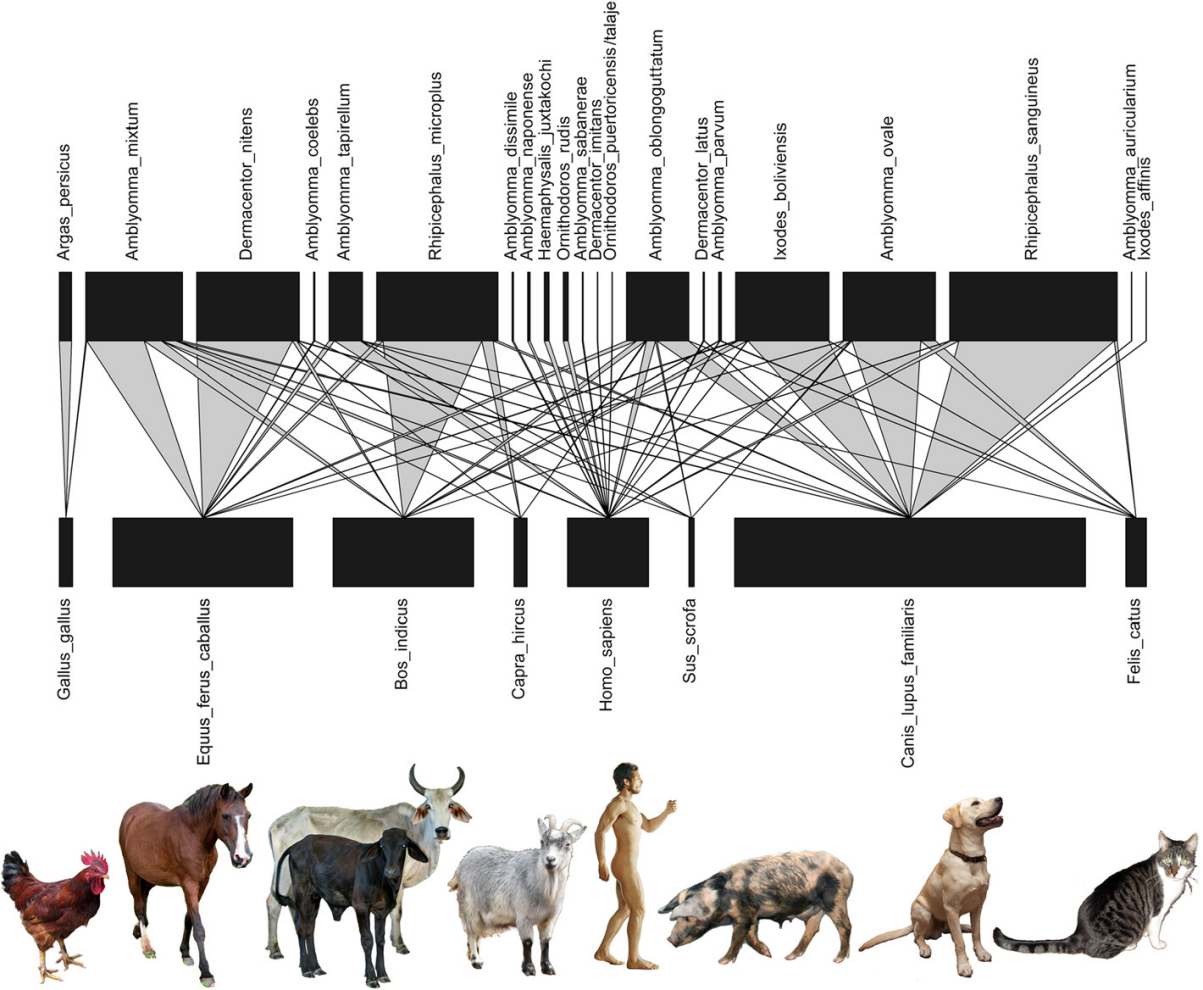
Host associations of ticks with domestic animals and humans, visualized by a bipartite network. Nodes (*black*) represent species and links (*grey*) correspond to species interactions. Variation in interaction frequencies are reflected by the width of the links. The network is arranged such that it shows minimal crossings of interactions, which allows for easier interpretation

A total of 14 different tick species were recorded from seven species of domestic animals (see Additional file 4: Table S1). The tick species most often associated with poultry, horse, cattle and dog are globally recognized as economically important pests of these host species, i.e. *Argas persicus* (poultry tick), *Dermacentor nitens* (tropical horse tick), *Rhipicephalus microplus* (southern cattle tick), and *Rhipicephalus sanguineus* (brown dog tick), respectively (Fig. 4). Other ticks that were commonly found on domestic animals include *Amblyomma mixtum* (part of the *A. cajennense* species complex), which was predominantly collected from horses, *Amblyomma ovale* and *Ixodes boliviensis*, which were most abundant on dogs, and *Amblyomma oblongoguttatum*, a more generalist tick that was found on all domestic animals except poultry. The remaining tick species (*Amblyomma auricularium*, *Amblyomma coelebs*, *Amblyomma parvum*, *Amblyomma tapirellum*, *Dermacentor latus*, *Ixodes affinis*) were infrequently collected from domestic animals and their records may represent incidental infestations. In addition, a total of 15 different tick species parasitized humans, of which *Amblyomma tapirellum* was most often involved (Fig. 4).

## Discussion

Our results indicate that the majority of tick species in our study system showed significant structural and/or phylogenetic specificity during the adult life stage, regardless of the spatial scale considered. Thus, adult ticks used some host species disproportionally more than others, and host species tended to be phylogenetically closely related. This specificity was found at the host species-, family- and order-level, with only few tick species having substantial adult tick records from multiple host orders. Moreover, more diverse tick genera parasitized more diverse host species, suggesting that distinct tick species have specialized on different host species. While most tick species were specialists of phylogenetically closely related host species, these host species in turn were parasitized by ticks from different genera, resulting in asymmetric tick-host phylogenetic interactions.

Our findings are consistent with empirical studies of other host-parasite systems, including helminths, chewing lice and fleas parasitic on small mammals. These studies indicated that most parasites are highly host-specific to a limited number of host species [54], and that host specificity tends to be phylogenetically constrained [19, 55] and geographically scale-invariant [9]. A recent analysis of flea-mammal networks showed that closely related host species shared the same flea species, but that these fleas belonged to different lineages [56]. This pattern is similar to that observed in our study and can be explained by processes such as host-switching, ecological fitting and/or co-evolutionary alternation [56–58]. Further, McCoy et al. [8] reported a positive correlation between the number of African tick species within a given genus and their recorded number of hosts, as we did here for Neotropical ticks. The high specificity of parasite-host associations is likely a product of the continual coevolution of host defenses and parasite counter-defenses that should select for reciprocal specialization [57].

Several recent empirical studies have suggested that most tick species tend to be host generalists ([22–24, 59, 60], but see [33]). However, almost all of these studies also recognize that ticks show a continuous spectrum in specificity, ranging from the host species- to beyond the host order-level. In those cases, where tick species are not at either end of this spectrum, their classification as either host specialist or generalist can be somewhat subjective [21]. For example, Hoogstraal & Aeschlimann [33] considered tick species that feed exclusively from the class Reptilia (tortoises, snakes and lizards) to be strictly host-specific. In contrast, Nava & Guglielmone [23] classified ticks that feed on different host families and orders as generalists. This highlights the need for null models to evaluate whether obtained estimates of host specificity are significantly different from those predicted by the tick species’ expected probability distribution across its host species.

Although we did not find any spatial scaling of host specificity, such pattern may still exist across larger geographical scales. Most tick species in our study system have geographical distributions that extend beyond Panama [13], and our results can therefore not be generalized across the entire range of these species. For example, tick species that were either exclusively (i.e. *A. longirostre*, *A. pecarium* and *H. leporispalustris*) or primarily (i.e. *A. coelebs* and *D. nitens*) associated with one particular host species in Panama, were shown to feed from a variety of host species and families across their entire range [23]. Likewise, tick species that exclusively (i.e. *A. nodosum*) or primarily (i.e. *A. auricularium*, *A. calcaratum* and *A. pacae*) parasitized one particular host family in Panama, were specific at the host order-level across their entire range [23]. Thus, whereas we found high phylogenetic specificity within a relatively small portion of their range, Nava & Guglielmone [23] found that these tick species exhibited much lower phylogenetic specificity across their Neotropical distribution, throughout which the spectrum of potential host species is much larger. The hypothesis that ticks may be “local specialists but global generalists” [8], may therefore still hold for these species. However, recent discoveries of cryptic species among tick populations from different geographical areas (e.g. *A. cajennense* [29], *A. parvum* [61], *R. microplus* [62] and *R. sanguineus* [63]), as well as experimental evidence for host-associated genetic races [1, 64, 65], stresses the need for considering tick population genetic structure in future studies, particularly when large geographical areas are considered.

Very few tick species in our study system can be considered host generalists in the broadest sense, i.e. by using host species in proportion to their availability (lack of structural specificity) while at the same time feeding from distantly related host species (lack of phylogenetic specificity). For example, *A. mixtum* (part of the *A. cajennense* species complex) parasitized 16 species of wild and domestic hosts in natural and anthropogenic environments, yet nearly half of our records involve horses. Hence, host specificity in this species was structurally low, but phylogenetically high. Other empirical studies have also revealed that apparent generalist tick species may show local host preferences [8, 64, 65], which illustrates the complementarity and importance of considering both structural and phylogenetic aspects of host specificity [25].

Domestic animals were principally parasitized by tick species that are globally recognized as important economic pests and which were able to spread to Panama following the introduction of their domestic hosts [12]. Only few native tick species were frequently collected from domestic animals. Perhaps not surprisingly, these ticks are known to feed from a wide variety of natural host species [13], although they too tended to show a structural and phylogenetic bias. Specifically, *A. ovale* and *I. boliviensis* infested dogs in high numbers but were principally associated with wild carnivores. Likewise, *A. mixtum* and *A. oblongoguttatum* parasitized no less than nine different host orders, the largest number for all tick species in our dataset, but the former was most often found on odd-toed ungulates (particularly horses) while the latter chiefly fed from carnivores (particularly canids) and, to a lesser extent, ungulates (including horses and cattle). Overall, our results suggest that, probably with the exception of *A. mixtum*, domestic animals are not important host species for most of the native tick species in Panama.

Highly specific tick-host relationships as observed in our study have implications for tick-borne disease transmission. On the one hand, high host specificity limits the potential routes for interspecific pathogen transmission, thereby decreasing the risk for emerging infectious diseases. On the other hand, our findings that closely related hosts are parasitized by distantly related ticks, suggest that host switching events frequently occurred throughout the life history of these ticks. Previous empirical studies have also shown that ticks can switch hosts under changing environmental conditions, such as climate change, host availability, or even acaricide use [1, 8, 66]. In fact, host switching has been suggested to be ubiquitous for many parasites at both evolutionary and ecological time scales [58]. Current ecological perturbations and human activities should only facilitate the potential for host switching, which in turn may increase the risk for tick-borne pathogen transmission between hosts, including livestock, pets and humans [58].

Despite the robustness of the specificity indices we used, our analyses and inferences do have limitations that are inherent to all studies based on field observations and published datasets. First, more intensive sampling would likely provide new tick-host associations, potentially lowering host specificity estimates for some tick species. However, as we corrected for differences in sampling effort, we do not expect the overall conclusions to be profoundly affected. Moreover, using four additional network indices to measure structural specificity, the results remained the same: host specificity is high for the adult ticks in Panama (see Additional file 2: Supplementary analyses, Figure S1, Table S2). Secondly, studies based on field collections are usually unable to differentiate between failed and successful feeding events. Experiments are needed that assess differential tick performance on various host species to support field-based evidence for host specificity [1, 67]. Thirdly, with the continual discovery of species complexes there is a need for genetic data to determine whether perceived “generalists” may in fact consist of multiple cryptic “specialist” species [8, 64].

Another important aspect to consider is the potential differences in feeding relationships between larvae, nymphs and adult ticks. While larvae and nymphs only feed from vertebrates for their development, the adults of many tick species also search for a mating partner on a host, which may drive specificity in adults but generality in immature stages [22]. Unfortunately, the host associations and in some cases morphological descriptions of immature ticks are poorly documented for Panama, so that we had to limit our study to adult ticks. Empirical studies from elsewhere in the Neotropics suggest that the immature forms of three-host ixodid ticks may feed from entirely different host groups [13] and that they tend to be less host-specific [23] than their adult counterparts. It thus seems reasonable to expect that the host-use patterns of immature ticks in Panama differ from those of the adult stage. An important question is whether these larvae and nymphs are true host generalists, or rather specific to different groups of host species compared to the adult stage. This knowledge is imperative for predicting environmental impacts, such as cascade effects of biodiversity loss on tick populations and/or disease transmission. More complex life-cycles in combination with high host specificity increase the risk of local parasite-host coextirpation [7]. Thus, if different life stages are specific to different host species, loss of host diversity should cause stronger bottleneck events compared to a situation where only the adult life stage is host-specific. Future studies that focus on ontogenetic shifts in tick-host relationships are therefore warranted.

It is important to stress that our results do not rule out the possibility that some tick species are more constrained by adaptations to environmental conditions than by host adaptation. Many tick species spend the majority of their life-cycle off-host, so that both abiotic (climatic) and biotic (host) factors determine tick distribution, abundance and host relationships [68]. Cumming [59] already showed that the range limits of most African tick species are not limited by their host species’ distribution, suggesting that environmental factors may be more important. In Panama, environmental specificity of ticks plays an important role in the life history of the Argasidae. These so-called “endophilic” tick species are confined to caves, burrows, roosts and other habitats where host species gather in large numbers and/or regularly return to [60]. Indeed, the *Ornithodoros* ticks in our study were among the least host-specific species. Some of the ixodid ticks in Panama also show clear environmental preferences. For example, certain species of *Dermacentor* and *Ixodes* seem to be restricted to wetter, montane environments [27, 30]. On the other hand, these particular habitats are characterized by extraordinary vertebrate diversity, yet the adult ticks of these species still predominantly feed from a limited number of closely related host species. This suggests that both environmental adaptations and host adaptations may act in concert to shape the specific tick-host relations observed in Panama.

Future experimental studies may reveal the relative importance of environmental conditions *versus* host suitability for explaining the highly specific tick-host relationships that we found in our study. Specifically, to what extent do the realized host relationships that were observed match potential host relationships if abiotic factors were irrelevant? Experimental studies have so far demonstrated that many tick species are able to complete their life-cycles on laboratory animals that are phylogenetically distant from their natural host species [69–71]. This suggests that the potential host specificity of ticks may be lower than their realized specificity. However, a substantial body of evidence also suggests that tick physiological processes, such as molting, engorgement, hatching, oviposition, and even survival, are negatively affected when ticks are fed on unnatural host species [1, 67, 70, 72–77]. These studies also showed that tick fitness was higher on laboratory animals that were phylogenetically more closely related to the tick species’ natural host species. Clearly, there is a need for better integration of both field-based and experimental studies to increase our understanding of tick-host specificity [65, 67].

## Conclusions

Our findings indicate that most tick species in Panama are scale-invariant host specialists during the adult life stage. This implies high vulnerability to local tick-host coextirpation [7] so that any reduction of host diversity will lead to impoverished tick communities that are dominated by generalist tick species [43]. These persistent generalist species may be instrumental in tick-borne disease dynamics as they bear the highest potential for widespread pathogen transmission across host species in local communities [59, 65]. Host extinction may, therefore, more likely increase rather than limit the risk of tick-borne disease outbreaks [59]. Future studies should investigate how alterations of tick-host network properties due to anthropogenic disturbances affect disease dynamics, particularly in tropical regions where wildlife diversity is rapidly eroding [15].

## Abbreviations

MPD, mean phylogenetic distance; NRI, Net Relatedness Index/Nearest Relative Index; SES_MPD,_ standardized effect size of MPD

## Additional files

Additional file 1: Table S1. Summary of data on ticks, their vertebrate hosts, and the area for each spatial scale. *Abbreviations*: TS, number of tick species; HS, number of host species; THA, number of tick-host associations; SL, number of species links (non-zero entries in the matrix). (DOCX 1997 kb) Additional file 2: Supplementary analyses. In addition to the network-level (*H*_2_^'^) and species-level (*d*_*i*_^'^) specificity indices, we used four other quantitative metrics (Table S1) to test whether the structural specificity of tick-host communities in Panama is significantly higher than predicted by null models I and II. **Figure S1.** Estimates of generality, vulnerability, interaction evenness and modularity (black dots) of tick-host interaction networks were significantly different from those predicted by null model I (grey boxplots) and null model II (dashed boxplots) at each spatial scale **Table S1**. Summary of the network metrics considered in this study **Table S2**. Network properties of tick-host associations after network rarefaction of the large and intermediate scales. (DOCX 36 kb) Additional file 3: Figure S1. Phylogenetic tree of vertebrate host species in our dataset. (DOCX 2322 kb) Additional file 4: Table S1. Database of tick-host associations used for the analyses of this study. (XLSX 39 kb)
