# Supplementary material for: Host specificity in a diverse Neotropical tick community: an assessment using quantitative network analysis and host phylogeny
Source: Parasit Vectors. 2016 Jun 29;9:372. doi: 10.1186/s13071-016-1655-6 (PMC4928246; doi:10.1186/s13071-016-1655-6)
Supplement: Additional file 1: Table S1. — Summary of data on ticks, their vertebrate hosts, and the area for each spatial scale. Abbreviations: TS, number of tick species; HS, number of host species; THA, number of tick-host associations; SL, number of species links (non-zero entries in the matrix). (DOCX 1997 kb) [file 13071_2016_1655_MOESM1_ESM.docx]

## Additional file 1: Table S1 Summary of data on ticks, their vertebrate hosts and the area for each spatial scale. *Abbreviations*: TS, number of tick species; HS, number of host species; THA, number of tick-host associations; SL, number of species links (non-zero entries in the matrix)

|  | **TS** | **HS** | **THA** | **SL** | **Area (km^2^)** |
| --- | --- | --- | --- | --- | --- |
| **Spatial scale** |  |  |  |  |  |
| **Large** | 41 | 68 | 5,298 | 207 | 74,340 |
| **Medium** | 28 | 57 | 4,037 | 153 | 59,710 |
| **Small** | 25 | 44 | 2,666 | 111 | 2,178 |
| **Data source** |  |  |  |  |  |
| **Unpublished** | 20 | 21 | 1,159 | 59 | – |
| **Published^[1-13]^** | 40 | 66 | 4,139 | 191 | – |

**References**

1. Apanaskevich DA, Bermúdez SE. Description of a new *Dermacentor* (Acari: Ixodidae) species, a parasite of wild mammals in Central America. J Med Entomol*.* 2013;50(6):1190–1201.

2. Bermúdez SE, Eremeeva ME, Karpathy SE, Samudio F, Zambrano ML, Zaldivar Y, et al. Detection and identification of rickettsial agents in ticks from domestic mammals in eastern Panama. J Med Entomol*.* 2009;46(4):856–861.

3. Bermúdez SE, Miranda RJ, Smith D. Tick species (Ixodida) in the Summit Municipal Park and adjacent areas, Panama City, Panama. Exp Appl Acarol*.* 2010;52(4):439–448.

4. Bermúdez SE, Zaldivar Y, Spolidorio MG, Moraes-Filho J, Miranda RJ, Caballero CM, et al. Rickettsial infection in domestic mammals and their ectoparasites in El Valle de Antón; Coclé; Panamá. Vet Parasitol. 2011;177(1–2):134–138.

5. Bermúdez S, Miranda R, Zaldívar Y, González P, Berguido G, Trejos D, et al. Detection of *Rickettsia* in ectoparasites of wild and domestic mammals from the Cerro Chucanti private reserve and from neighboring towns, Panama, 2007–2010. Biomedica 2012;32(2):189–195.

6. Bermúdez SE, Castro A, Esser H, Liefting Y, García G, Miranda RJ. Ticks (Ixodida) on humans from central Panama, Panama (2010–2011). Exp Appl Acarol*.* 2012;58(1):81–88.

7. Bermúdez S, Esser HJ, Miranda R, Moreno R. Wild carnivores (Mammalia) as hosts for ticks (Ixodida) in Panama. Syst Appl Acarol*.* 2015;20(1):13–19.

8. Bermúdez SE, Torres S, Aguirre Y, Domínguez L, Bernal Vega JA. A review of *Ixodes* (Acari: Ixodidae) parasitizing wild birds in Panama, with the first records of *Ixodes* *auritulus* and *Ixodes bequaerti*. Syst Appl Acarol*.* 2015;20(8):847–853.

9. Dunn LH. The ticks of Panama, their hosts, and the diseases they transmit. Am J Trop Med Hyg*.* 1923; S1–3(2):91–104.

10. Dunn LH. Ticks from tapirs of Panama. J Parasitol*.* 1934;20(5):312–312.

11. Fairchild GB. An annotated list of the bloodsucking insects, ticks and mites known from Panama. Am J Trop Med*.* 1943;S1–23(6):569–591.

12. Fairchild GB, Kohls GM, Tipton VJ. The ticks of Panama (Acarina: Ixodidea). In: Wenzel RL, Tipton VJ, editors. Ectoparasites of Panama*.* Field Museum of Natural History, USA: Chicago; 1966. p. 167–219.

13. García GG, Castro A, Rodriguez I, Bermúdez SE. Ixodid ticks of *Hydrochaeris isthmius* Goldman 1912 (Rodentia: Caviidae) in Panama. Syst Appl Acarol*.* 2014;19(4):404–408.
