# Supplementary material for: Host specificity in a diverse Neotropical tick community: an assessment using quantitative network analysis and host phylogeny
Source: Parasit Vectors. 2016 Jun 29;9:372. doi: 10.1186/s13071-016-1655-6 (PMC4928246; doi:10.1186/s13071-016-1655-6)
Supplement: Additional file 2: — Supplementary analyses. In addition to the network-level (H 2') and species-level (d i') specificity indices, we used four other quantitative metrics (Table S1) to test whether the structural specificity of tick-host communities in Panama is significantly higher than predicted by null models I and II. Figure S1. Estimates of generality, vulnerability, interaction evenness and modularity (black dots) of tick-host interaction networks were significantly different from those predicted by null model I (grey boxplots) and null model II (dashed boxplots) at each spatial scale Table S1. Summary of the network metrics considered in this study Table S2. Network properties of tick-host associations after network rarefaction of the large and intermediate scales. (DOCX 36 kb) [file 13071_2016_1655_MOESM2_ESM.docx]

**Additional file 2: Supplementary analyses.** In addition to the network-level ($H_{2}^{'}$) and species-level ($d_{i}^{'}$) specificity indices, we used four other quantitative metrics (Table S1) to test whether the structural specificity of tick-host communities in Panama is significantly higher than predicted by null models I and II:

**(1)** Generality ($G_{qw}$); defined as the reciprocal of the Shannon diversity of links for the highest trophic level, this index reflects the effective mean number of host species per tick species, weighted by their marginal totals. The higher $G_{qw}$ for tick species *i*, the less specific it is, i.e. the more host species it parasitizes. Originally developed by Bersier et al. [1], who used base 2 logarithms in their equations, we based equations on ln.

**(2)** Vulnerability ($V_{qw}$); Like $G_{qw}$ but now for the lowest trophic level, this index reflects the effective mean number of tick species per host species, weighted by their marginal totals [1]. The higher $V_{qw}$ for host species *j*, the less specific it is, i.e. the more tick species it supports and thus the larger its importance for the overall tick community.

**(3)** Interaction Evenness ($E_{2}$); based on the Shannon diversity of all possible links, this index expresses how homogeneously the tick and host species are connected [2]. Networks whose distribution of observed interaction frequencies is highly heterogeneous will have $E_{2}$ values close to 0. In contrast, $E_{2}$ values close to 1 imply well-connected networks in which tick species interact with the available host species with similar frequencies. Rather than using the sum of only the realized links [3], we used the product of the matrix dimensions (i.e. all potential links) to determine $E_{2}$ [4] (for more details, see the manual of the package ‘bipartite’, version February 19, 2015 [5]).

**(4)** Modularity (*Q*); this index quantifies the presence of cohesive groups called “modules”, in which species are linked better within than across modules. To estimate the level of modularity and the number of modules within the network, we used the QuaBiMo-algorithm, which allows for the use of weighted (quantitative) links and computes modularity *Q* using a Markov Chain Monte Carlo approach [6]. A total of 10^8^ MCMC steps were used with a tolerance level of 10^-10^. *Q* ranges from 0 for random networks to 1 for perfectly modular networks. High modularity suggests specificity of ticks for certain groups of hosts that in turn do not support many other species of ticks.

We found that generality, vulnerability and interaction evenness were significantly lower, and that modularity was significantly higher, than predicted by both null models (*P* = 0.000). Rarefaction had negligible effects on each of the metrics’ values and there was no clear trend among rarefied values of *Q*, $E_{2}$, $G_{qw}$ and $V_{qw}$ across the spatial scales (Table S2). The high *Q* estimates suggest high structural specificity towards particular groups of hosts and that these “modules” of interacting tick and host species had few connections to other modules. The low $E_{2}$estimates reveal high heterogeneity in interaction frequencies and low connectivity between species of ticks and vertebrate hosts. The low $G_{qw}$ and $V_{qw}$ estimates indicate that ticks parasitized very few host species and hosts were parasitized by very few tick species compared to null-model expectations. These results are in agreement with the estimates of $H_{2}^{'}$ and$d_{i}^{'}$, corroborating that the host associations of adult ticks in Panama tend to be highly specific.

**Additional file 2: Fig. S1** Estimates of generality, vulnerability, interaction evenness and modularity (black dots) of tick-host interaction networks were significantly different from those predicted by null model I (grey boxplots) and null model II (dashed boxplots) at each spatial scale

**Additional file 2: Table S1** Summary of the network metrics considered in this study

| **Level** | **Metric** | **Definition** | **Formula** | **Reference** |
| --- | --- | --- | --- | --- |
| **Species** |  |  |  |  |
| $\boldsymbol{d}_{\boldsymbol{i}}^{\boldsymbol{'}}$ | Standardized Kullback-Leibler distance | Degree of interaction specialization of species *i* | $d_{i}=\sum_{j=1}^{J} \left( p_{ij}^{'}\cdot\ln\frac{p_{ij}^{'}}{q_{j}} \right)$ , which is normalized to  $d_{i}^{'}=\frac{d_{i}-d_{min}}{d_{max}-d_{min}}$ | [7] |
| **Network** |  |  |  |  |
| $\boldsymbol{H}_{\boldsymbol{2}}^{\boldsymbol{'}}$ | Standardized Interaction Diversity | Degree of interaction specialization among ticks and hosts of the entire network | $H_{2}=-\sum_{i=1}^{I} \sum_{j=1}^{J} {(p}_{ij}\cdot\ln p_{ij})$, which is normalized to  $H_{2}^{'}=\frac{H_{2max}-H_{2}}{H_{2max}-H_{2min}}$ | [7] |
| $\boldsymbol{E}_{\boldsymbol{2}}$ | Interaction Evenness | Evenness of interactions in the network | $E_{2}=H_{2}/\ln IJ$ | [4] |
| $\boldsymbol{G}_{\boldsymbol{qw}}$ | Generality | Weighted mean effective no. of host species per tick species | $G_{qw}=\sum_{j=1}^{J} {\frac{A_{j}}{N}e}^{H_{j}}$ | [1] |
| $\boldsymbol{V}_{\boldsymbol{qw}}$ | Vulnerability | Weighted mean effective no. of tick species per host species | $V_{qw}=\sum_{i=1}^{I} {\frac{A_{i}}{N}e}^{H_{i}}$ | [1] |
| ***Q*** | Modularity | Degree of network partitioning into modules of highly connected species | $Q=\frac{1}{2N}\sum_{ij} (A_{ij}-K_{ij})\delta(m_{i},m_{j})$ | [6] |

Note: metrics were calculated over contingency tables with *I* rows of host species and *J* columns of tick species; the number of observed interactions between host species *i* and tick species *j* was defined as$a_{ij}$; *N* is the total number of observed interactions for the entire web; *L* is the number of all realized links;$A_{i}$ and $A_{j}$ are respectively the total number of interactions in which *i* and *j* were involved (i.e. the respective row and column totals); $p_{ij}$ is defined as $a_{ij}$ in relation to *N*; $p_{ij}^{'}$ is the proportion of $a_{ij}$ in relation to $A_{i}$; $q_{i}$ and $q_{j}$ was defined as respectively $A_{i}$ and $A_{j}$ in relation to N*;*$A_{ij}$ and $K_{ij}$ are respectively the normalized interaction matrix and null model matrix, where the sum of all link strengths equals 1; $m_{i}$ and $m_{j}$ are the modules to which species *i* and *j* are respectively assigned; the indicator function $\delta(m_{i},m_{j})$ equals 1 when $m_{i}=m_{j}$ and is otherwise equal to zero.

**Additional file 2: Table S2** Network properties of tick-host associations after network rarefaction of the large and intermediate scales. Standard errors (SE) of rarefied values were all smaller than 0.006

|  | **Geographic scale** | | |
| --- | --- | --- | --- |
| **Metric** | **Large** | **Intermediate** | **Small** |
| **Network-level** |  |  |  |
| Interaction evenness ($E_{2}$) | 0.52 | 0.52 | 0.53 |
| Vulnerability ($V_{qw}$) | 2.96 | 2.44 | 2.55 |
| Generality ($G_{q\boldsymbol{w}}$) | 3.26 | 3.37 | 2.89 |
| Modularity (*Q*) | 0.70 | 0.70 | 0.74 |

## References

1. Bersier L-F, Banašek-Richter C, Cattin M-F. Quantitative descriptors of food-web matrices. Ecology 2002;83(9):2394–2407.

2. Blüthgen N, Fründ J, Vázquez DP, Menzel F. What do interaction network metrics tell us about specialization and biological traits? Ecology 2008;89(12):3387–3399.

3. Tylianakis JM, Tscharntke T, Lewis OT. Habitat modification alters the structure of tropical host-parasitoid food webs. Nature 2007;445:202–205.

4. Dormann CF, Fründ J, Blüthgen N, Gruber B. Indices, graphs and null models: analyzing bipartite ecological networks. Open Ecol J. 2009;2:7–24.

5. Dormann CF, Gruber B, Fründ J. Introducing the bipartite package: analysing ecological networks. R News 2008;8(2):8–11.

6. Dormann CF, Strauss R. A method for detecting modules in quantitative bipartite networks. Methods Ecol Evol. 2014;5(1):90-98

7. Blüthgen N, Menzel F, Blüthgen N. Measuring specialization in species interaction networks. BMC Ecology 2006;6(1):9.
