# Supplementary material for: Host specificity in a diverse Neotropical tick community: an assessment using quantitative network analysis and host phylogeny
Source: Parasit Vectors. 2016 Jun 29;9:372. doi: 10.1186/s13071-016-1655-6 (PMC4928246; doi:10.1186/s13071-016-1655-6)
Supplement: Additional file 3: Figure S1. — Phylogenetic tree of vertebrate host species in our dataset. (DOCX 2322 kb) [file 13071_2016_1655_MOESM3_ESM.docx]

**Additional file 3. Figure S1.** Phylogenetic tree of vertebrate host species in our dataset. We used Wilson and Reeder [1] as taxonomic reference for Mammalia, supplemented by Voss and Jansa [2] for Didelphidae, Huchon and Douzery [3] for hystricognath rodents, Weksler [4] for muroid rodents, Johnson et al. [5] for Felidae, Bardeleben et al. [6] for Canidae, Koepfli et al. [7, 8] for Procyonidae and Mustelidae, Fry et al. [9], for Squamata, Lee [10] for Testudines and Jarvis et al. [11] for Aves.

**
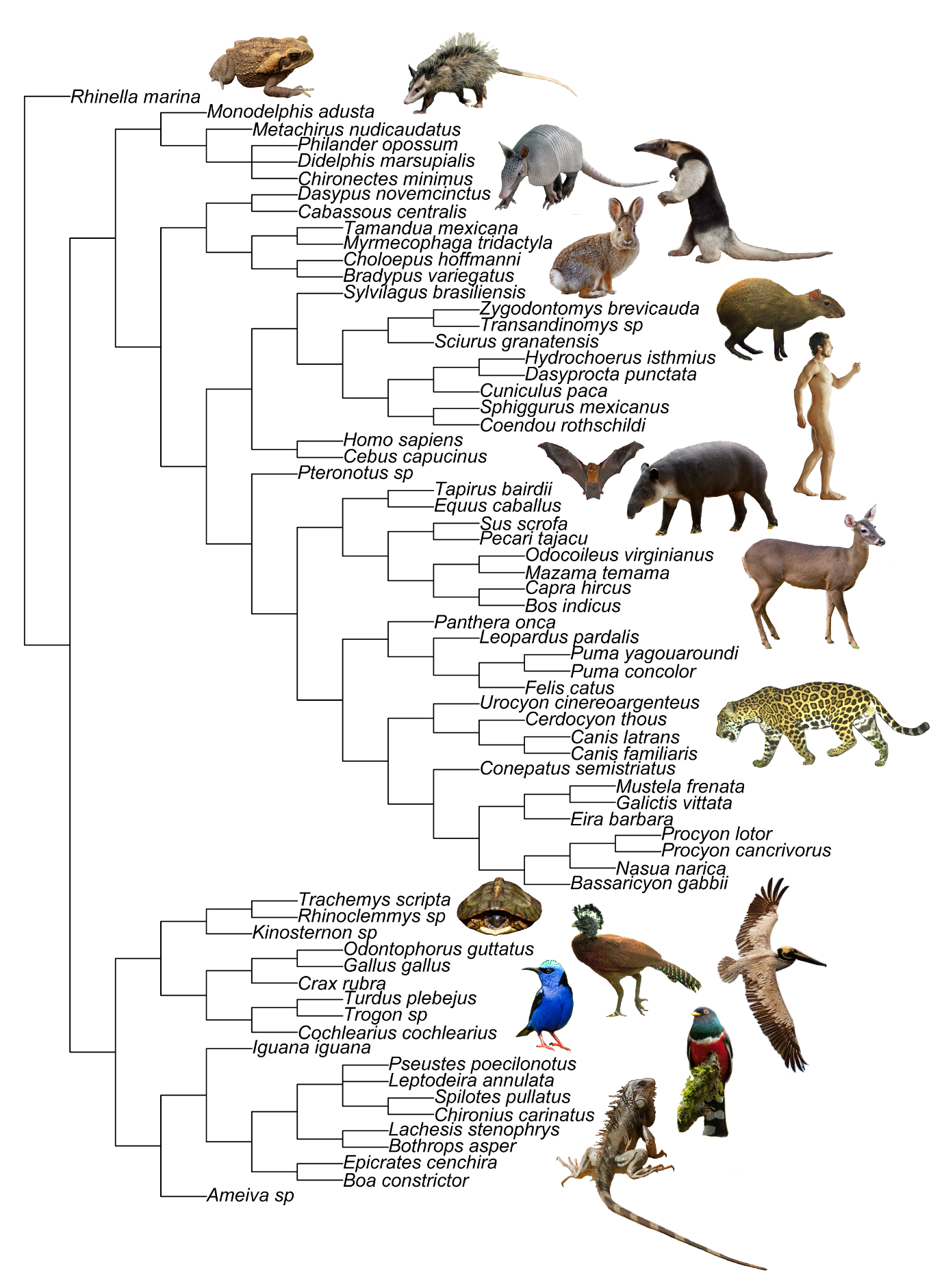
**

**References**

1. Wilson DE, Reeder DM. Mammal species of the world: a taxonomic and geographic reference, vol. 1, 3rd edn. Baltimore, Maryland, USA: John Hopkins University Press; 2005.

2. Voss RS, Jansa SA. Phylogenetic relationships and classification of didelphid marsupials, an extant radiation of New World metatherian mammals. Bull Am Mus Nat His. 2009:1–177.

3. Huchon D, Douzery EJP. From the Old World to the New World: a molecular chronicle of the phylogeny and biogeography of hystricognath rodents. Mol Phylogenet Evol. 2001;20(2):238–251.

4. Weksler M. Phylogenetic relationships of oryzomine rodents (Muroidea: Sigmodontinae): separate and combined analyses of morphological and molecular data. Bull Am Mus Nat His. 2006:1–149.

5. Johnson WE, Eizirik E, Pecon-Slattery J, Murphy WJ, Antunes A, Teeling E, et al. The late Miocene radiation of modern Felidae: a genetic assessment. Science 2006; 311(5757):73–77.

6. Bardeleben C, Moore RL, Wayne RK. A molecular phylogeny of the Canidae based on six nuclear loci. Mol Phylogenet Evol. 2005;37(3):815–831.

7. Koepfli KP, Gompper ME, Eizirik E, Ho CC, Linden L, Maldonado JE, et al. Phylogeny of the Procyonidae (Mammalia: Carnivora): molecules, morphology and the great American interchange. Mol Phylogenet Evol. 2007;43(3):1076–1095.

8. Koepfli K-P, Deere KA, Slater GJ, Begg C, Begg K, Grassman L, et al. Multigene phylogeny of the Mustelidae: resolving relationships, tempo and biogeographic history of a mammalian adaptive radiation. BMC Biology 2008;6(1):1–22.

9. Fry BG, Vidal N, Norman JA, Vonk FJ, Scheib H, Ramjan SFR, et al. Early evolution of the venom system in lizards and snakes. Nature 2006;439(7076):584–588.

10. Lee MSY. Turtle origins: insights from phylogenetic retrofitting and molecular scaffolds. J Evol Biol. 2013;26(12):2729–2738.

11. Jarvis ED, Mirarab S, Aberer AJ, Li B, Houde P, Li C, et al. Whole-genome analyses resolve early branches in the tree of life of modern birds. Science 2014; 346(6215):1320–1331.
